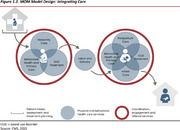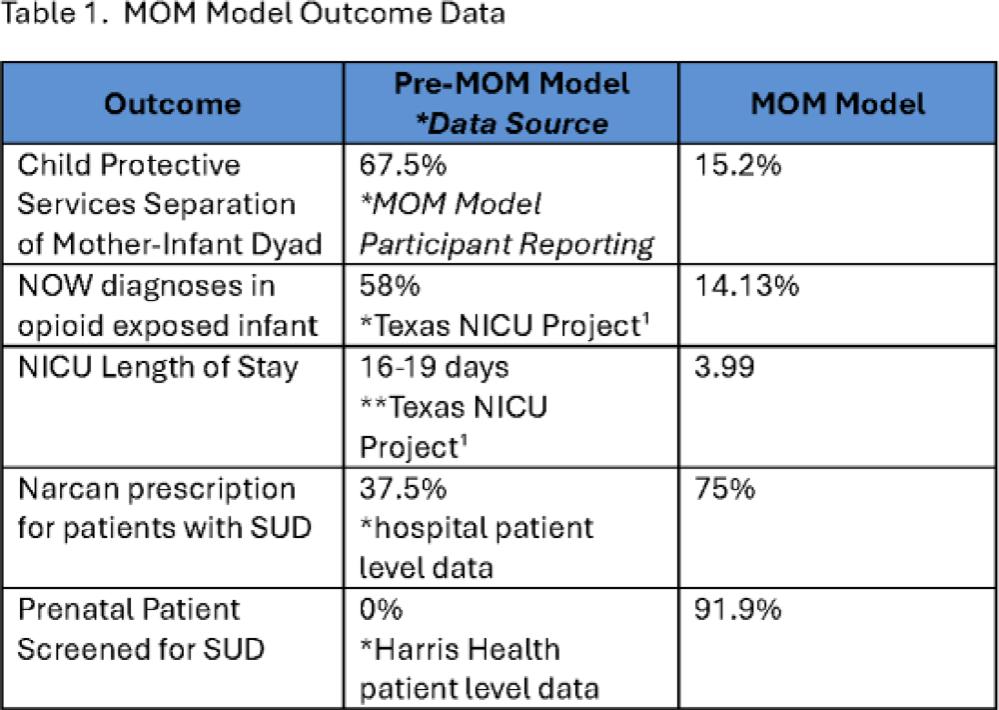# Oral Concurrent Session 9 – Quality: Friday, February 13, 2026 8:00 AM – 8:15 AM

**DOI:** 10.1002/pmf2.70151

**Published:** 2026-02-09

**8:00 AM – 8:15 AM**

## 90 | Reducing Obstetric Treatment Delays With Real‐Time Clinical Decision Support on Labor and Delivery

Nandini Raghuraman^1^; Megan L. Lawlor^2^; Lori M. Stevenson^3^; Steve Porter^4^; Susan Mann^5^; Amanda C. Zofkie^2^; Jeannie C. Kelly^1^; Antonina I. Frolova^2^; Roxane Rampersad^2^

^1^Washington University School of Medicine, Washington University School of Medicine, MO; ^2^Washington University School of Medicine, St. Louis, MO; ^3^Barnes‐Jewish Hospital, St. Louis, MO; ^4^Case Western Reserve University, University Hospitals MacDonald Women's Hospital, Cleveland, OH; ^5^Beth Israel Deaconess Medical Center, Harvard Medical School, Boston, MA

**Objective**: Timely treatment of severe hypertension (HTN) and intraamniotic infection (IAI) are key quality metrics for labor and delivery (L&D) units. We evaluated whether riskLD, a clinical decision support tool integrated within electronic health records (EHR) which issues real‐time alerts to prompt timely care, reduced treatment delays.

**Study Design**: We conducted an interrupted time series study over 24 months (3/2023‐2/2025) on a single tertiary care L&D unit. riskLD was implemented in February 2024. We assessed monthly rates of delayed initiation of antibiotics for IAI (>1 hour) after meeting ACOG‐defined IAI criteria and delayed treatment of severe HTN (>1 hour) after two consecutive severe blood pressures. Segmented regression was used to assess trends over time. Prais–Winsten estimation was used to account for autocorrelation. Models included terms for pre‐implementation trend, immediate change after riskLD implementation, and post‐implementation trend.

**Results**: There were 2992 and 3853 deliveries in the pre and post implementation periods, respectively. For IAI, there was a significant increasing trend in delayed treatment prior to riskLD implementation (β  =  +3.7% per month, *p*  =  0.01), followed by a significant immediate 32% reduction in delayed treatment after riskLD implementation (*p*  =  0.01) that remained stable post‐implementation (*p* = 0.24). For severe HTN, there was no significant trend pre‐implementation, however riskLD implementation was associated with a significant immediate 9% reduction in treatment delays (*p*  =  0.048), which remained stable post‐implementation (*p* = 0.40).

**Conclusion**: Implementation of real‐time EHR alerts via riskLD was associated with immediate and durable reductions in delayed treatment of IAI and severe HTN.

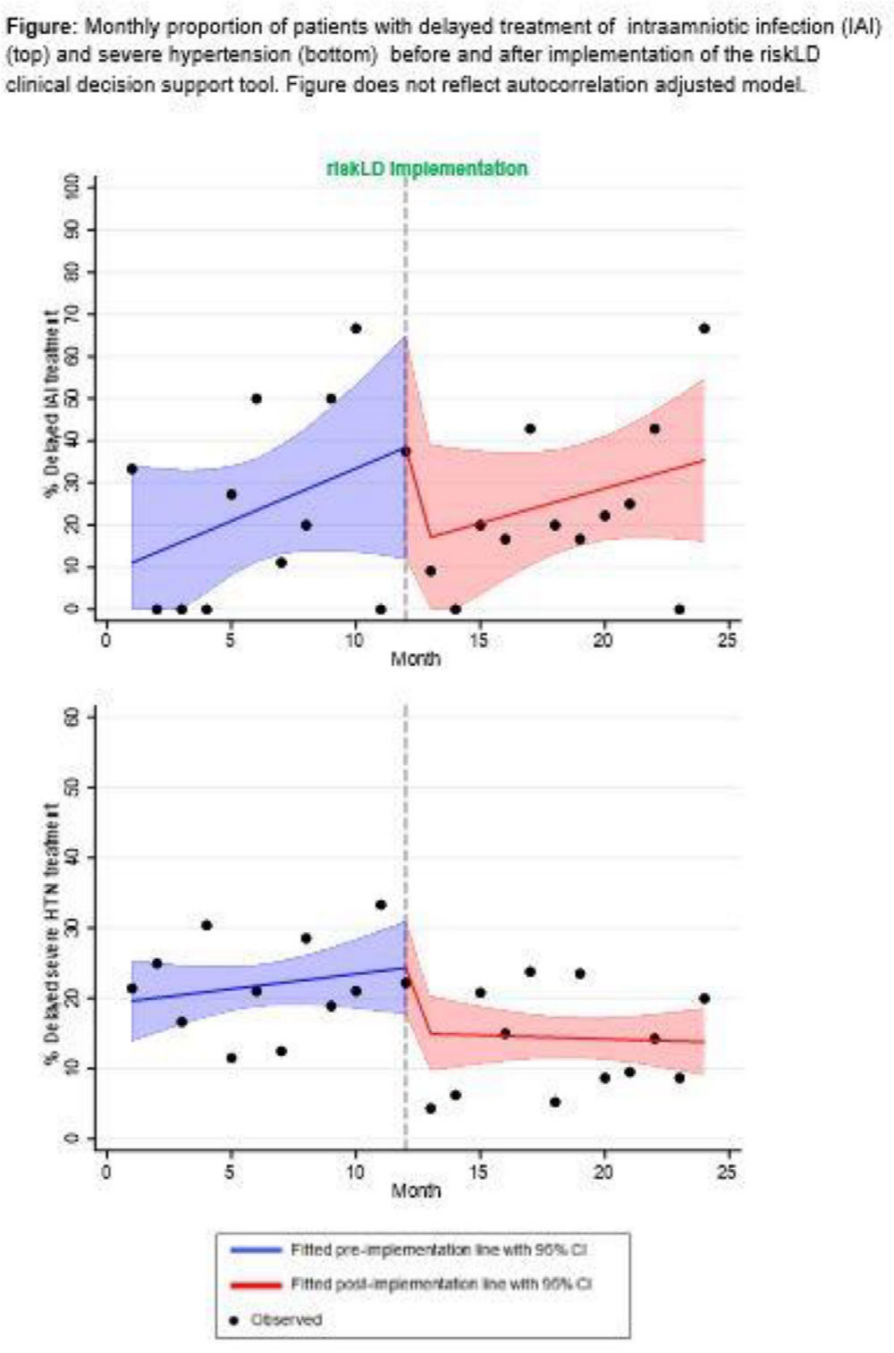

**8:15 AM – 8:30 AM**

## 91 | **Postpartum** Hemorrhage **Trends and Outcomes Before, During, and After a Statewide Obstetric Quality Improvement Effort**

Ellen Winter^1^; Timothy Wen^2^; Mary E. D'Alton^3^; Dena Goffman^1^; Alexander M. Friedman^1^

^1^Columbia University, New York, NY; ^2^UCSD, Irvine, CA; ^3^Division of Maternal‐Fetal Medicine, Department of Obstetrics and Gynecology, Columbia University Irving Medical Center, New York, NY

**Objective**: The NY Safe Motherhood Initiative (SMI), a statewide quality improvement effort, was convened in 2013 and included 117 of the 123 hospitals in NY State. A bundle to optimize management of obstetric hemorrhage was developed and disseminated. The purpose of this study was to evaluate trends in statewide postpartum hemorrhage (PPH) outcomes.

**Study Design**: Delivery hospitalizations in the 2007–2022 NY State Inpatient Database (SID) were analyzed for this repeated cross‐sectional analysis. The NY SID includes discharge data for all inpatient acute‐care hospitalizations in NY. For analyses, trends in PPH diagnoses among all delivery hospitalizations were first performed. Then, among deliveries with PPH, the following adverse outcomes were trended: (i) transfusion, (ii) non‐transfusion severe maternal morbidity (SMM), (iii) disseminated intravascular coagulation (DIC), and (iv) hysterectomy. Analyses were performed with joinpoint regression to determine the average annual percent change (AAPC).

**Results**: Among 3,693,509 delivery hospitalizations, PPH increased continuously from 2.2% in 2007 to 5.9% in 2022 (AAPC 6.9%, 95% CI 6.5%, 7.5%) (Figure 1). In joinpoint analysis, transfusion among deliveries with PPH increased from 2007 to 2013 (AAPC 2.1%, 95% CI 0.6%, 6.6%) but then decreased from 2013 to 2016 (AAPC –6.8%, 95% CI –9.5%, –2.1%) before increasing again from 2016 to 2022 (Figure 2). SMM increased from 2007 to 2014 (AAPC 2.8%, 95% CI 0.7%, 7.6%) before decreasing from 2014 to 2017 (AAPC –16.3%, 95% CI –20.8%, –8.4%), before rising again from 2017 to 2022 (AAPC 4.4%, 95% CI 0.1%, 18.5%). DIC increased from 2007 to 2014, decreased from 2014 to 2017 (AAPC –19.3%, 95% CI –25.3%, –8.9%) and increased non‐significantly from 2017 to 2022. Hysterectomy decreased significantly from 2013 to 2022 (AAPC ‐10.2%, 95% –14.3%, 8.7%).

**Conclusion**: The initiation of the NY SMI was associated with decreased risk for a range of adverse outcomes among deliveries with PPH. Decreases in risk continued for approximately 3‐4 years after initiation of the program with the exception of hysterectomy which decreased continuously.

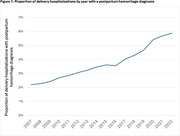

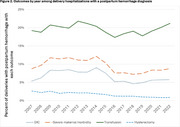

**8:30 AM – 8:45 AM**

## 92 | **The Impact of an Inpatient MFM Telemedicine Program on Maternal Morbidity**

Adina R. Kern‐Goldberger^1^; Cecilia Ganduglia Cazaban^2^; Chau Truong^2^; Sulki Park^2^; Sina Haeri^3^; Sindhu K. Srinivas^4^

^1^Cleveland Clinic Lerner College of Medicine, Cleveland, OH; ^2^UTHealth Houston School of Public Health, Houston, TX; ^3^Ouma Health, Austin, TX; ^4^Department of Obstetrics and Gynecology, Perelman School of Medicine, Maternal and Child Health Research Center, Philadelphia, PA

**Objective**: Maternal‐Fetal Medicine (MFM deserts) create geographic disparities in access to high‐risk obstetric care, adversely affecting outcomes. While telemedicine has expanded access in outpatient care, its impact in the inpatient setting is less understood. We evaluated whether an inpatient MFM telemedicine program was associated with reductions in severe maternal morbidity (SMM).

**Study Design**: This retrospective cohort study used the Texas Health Care Information Collection Inpatient Public Use Data File to analyze all deliveries at 3 Texas hospitals utilizing an MFM inpatient telemedicine. Program implementation occurred from November to December 2022, with January–June 2023 considered a wash‐in period. Outcomes during the post‐implementation period (July–December 2023) were compared with a matched pre‐implementation period (July–December 2022). The primary outcome was SMM, as defined by the CDC. Bivariable analyses evaluated patient characteristics, and multivariable logistic regression estimated adjusted odds of SMM.

**Results**: There were 2165 deliveries in the pre‐implementation cohort and 2253 post‐implementation. Table 1 depicts patient characteristics: post‐implementation had a higher proportion of patients <20 years old, Black patients, patients with commercial insurance, and higher mean obstetric co‐morbidity index. Adjusted analysis demonstrated a 34% reduction in SMM following program implementation (aOR 0.66, 95% CI 0.45–0.96, *p* = 0.03) (Table 2). The improvement in SMM was driven primarily by one hospital.

**Conclusion**: Implementation of an inpatient MFM telemedicine program was associated with significantly reduced SMM, highlighting its potential to improve outcomes in regions with limited access to MFM specialists. Future work should explore hospital‐level barriers and facilitators, as well as policy and reimbursement strategies to support broader adoption, equity, and scalability.

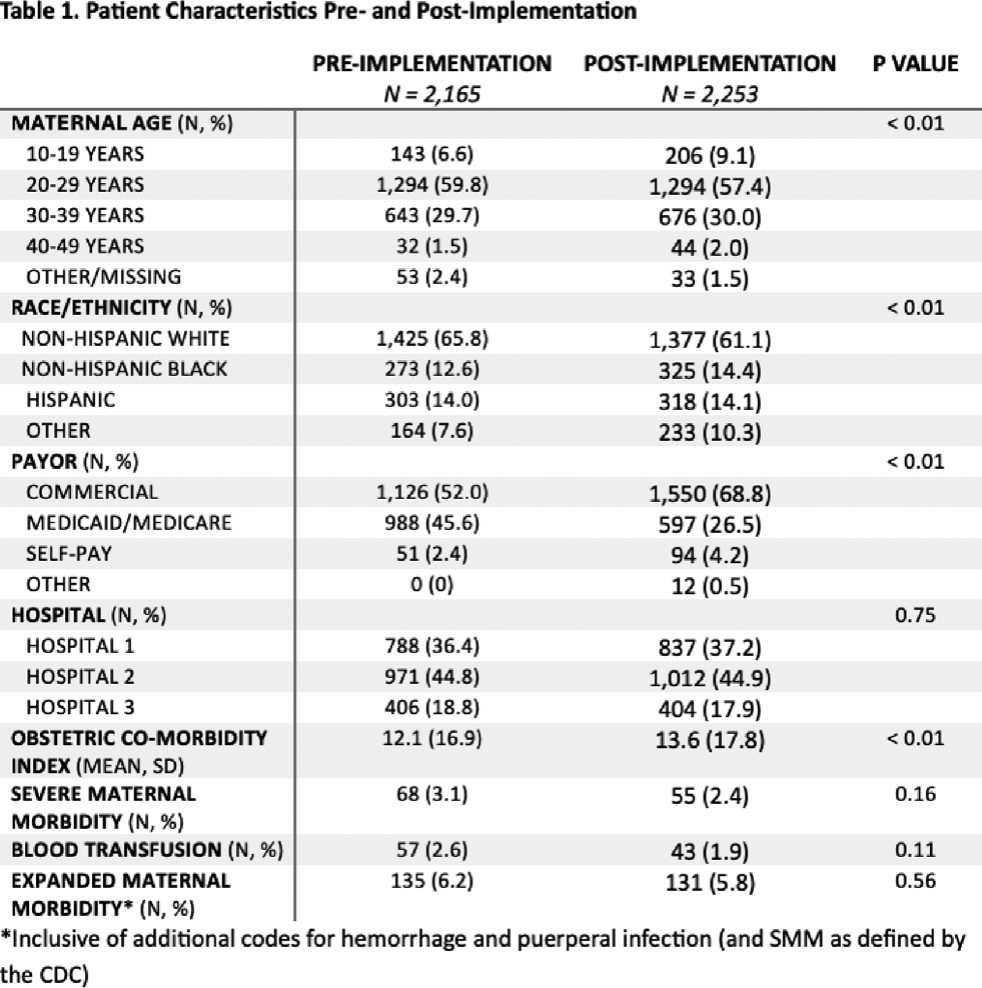

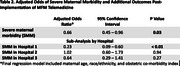

**8:45 AM – 9:00 AM**

## 93 | **Higher Hospital Volume Is Associated With Reduced Maternal Morbidity in Placenta Accreta Spectrum**

May Abiad^1^; Scott A. Shainker^2^; Karin A. Fox^3^; Anna M. Modest^4^; Giulia Bonanni^5^; George R. Saade^6^; Hiba J. Mustafa^7^; Lamia K. Atasi^8^; Albaro Nieto‐Calvache^9^; Rachel K. Harrison^10^; J. M. Newton^11^; Andrea L. Greiner^12^; Sonya S. Abdel‐Razeq^13^; Daniela A. Carusi^14^; Alissa R. Carver^15^; Christina M. Duzyj^14^; Brett D. Einerson^16^; Clarel Antoine^17^; Julie Kang^18^; Deirdre J. Lyell^19^; Amir A. Shamshirsaz^20^; Alfred Abuhamad^6^; Baha M. Sibai^21^; Robert M. Silver^16^; Alireza A. Shamshirsaz^22^

On behalf of the PAN‐AMERICAN SOCIETY FOR THE PLACENTA ACCRETA SPECTRUM (PAS2)

^1^Baptist Memorial Medical Education, Memphis, TN; ^2^Department of Obstetrics and Gynecology, Beth Israel Deaconess Medical Center, Harvard Medical School, Boston, MA; ^3^Division of Maternal Fetal Medicine, The University of Texas Medical Branch Health, Galveston, TX; ^4^Beth Israel Deaconess Medical Center, Boston, MA; ^5^Boston Children's Hospital, Boston, MA; ^6^Eastern Virginia Medical School at Old Dominion University, Norfolk, VA; ^7^Division of Maternal‐Fetal Medicine, Indiana University School of Medicine, Indianapolis, IN; ^8^Division of Maternal‐Fetal Medicine, Department of Obstetrics and Gynecology, Mercy Hospital, St. Louis, MO; ^9^Departamento de Ginecología y Obstetricia, Unidad de Alta Complejidad Obstétrica, Fundación Clínica Valle del Lili, Cali, Valle del Cauca; ^10^Department of Maternal‐Fetal Medicine, Advocate Aurora Health, Chicago, IL; ^11^Division of Maternal‐Fetal Medicine, Department of Obstetrics and Gynecology, Vanderbilt University Medical Center,, Nashville, TN; ^12^Department of Obstetrics and Gynecology, University of Iowa Hospitals and Clinics, Iowa City, IA; ^13^Division of Maternal‐Fetal Medicine, Department of Obstetrics, Gynecology, and Reproductive Sciences, Yale University, New Haven, CT; ^14^Department of Obstetrics and Gynecology, MassGeneral Brigham; Division of Maternal‐Fetal Medicine; Harvard Medical School, Boston, MA; ^15^Department of Obstetrics and Gynecology, Wilmington Maternal‐Fetal Medicine, Wilmington, NC; ^16^Department of Obstetrics and Gynecology, University of Utah Health, Salt Lake City, UT; ^17^Department of Obstetrics and Gynecology, New York University Grossman School of Medicine, New York, NY; ^18^Department of Maternal‐Fetal Medicine, Memorial Healthcare System, Tampa, FL; ^19^Division of Maternal‐Fetal Medicine, Department of Obstetrics and Gynecology, Stanford School of Medicine, Stanford, CA; ^20^Division of Maternal‐Fetal Medicine, Department of Obstetrics and Gynecology, Baylor College of Medicine, Houston, TX; ^21^Division of Maternal‐Fetal Medicine, Department of Obstetrics, Gynecology and Reproductive Sciences, McGovern Medical School at UTHealth Houston, Houston, TX; ^22^Boston Children's Hospital, Brigham and Women's Hospital, Beth Israel Deaconess Medical Center, Harvard Medical School, Boston, MA

**Objective**: To evaluate the relationship between hospital volume and maternal outcomes in patients with PAS and placenta previa, focusing on morbidity across low‐, medium‐, and high‐PAS‐volume centers.

**Study Design**: This retrospective observational study utilized the Nationwide Readmissions Database (2017–2019) to identify delivery hospitalizations with diagnoses of PAS and placenta previa using ICD‐10 codes. Hospitals were stratified into low‐ (≤3 PAS cases/year), medium‐ (4–12 cases/year), and high‐volume (≥13 cases/year) groups based on annual PAS case volume quartiles. The primary outcome was hemorrhage. Secondary outcomes included maternal transfusion, disseminated intravascular coagulation (DIC), shock, urinary system repair, and length of stay. A composite maternal morbidity score included hemorrhage, DIC, shock, blood transfusion, ventilation, and urinary system repair. Univariable and multivariable logistic regression models were used to assess the impact of hospital volume on maternal outcomes, adjusting for patient and hospital characteristics.

**Results**: Among 2165 patients with PAS and placenta previa, those managed at high volume hospitals experienced significantly lower rates of hemorrhage (57.2% vs. 67.4%, *p* = 0.002) and composite maternal morbidity without transfusion (63.0% vs. 70.3%, *p* = 0.036) compared to low volume hospitals. Blood transfusion rates were also lower at high volume hospitals (30.6% vs. 37.7%, *p* = 0.044). Multivariable analysis demonstrated that delivery at high volume hospitals was associated with reduced odds of hemorrhage (aOR 0.62, 95% CI 0.46–0.84) and transfusion (aOR 0.61, 95% CI 0.44–0.84) compared to low volume hospitals. Patients delivering at high volume hospitals also had significantly fewer severe complications, including DIC and shock. Length of stay from delivery to discharge remained consistent across groups.

**Conclusion**: High PAS‐volume hospitals demonstrated better maternal outcomes in patients with PAS and placenta previa, highlighting the benefits of multidisciplinary care with experienced teams in managing these high‐risk cases.

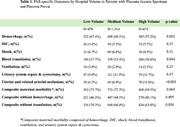

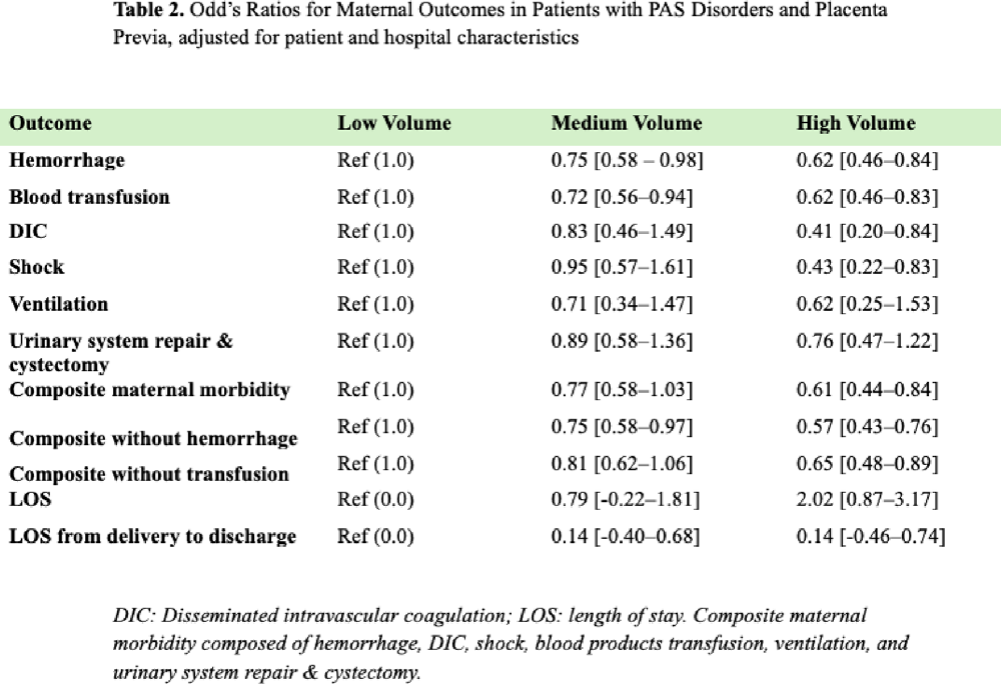

**9:00 AM – 9:15 AM**

## 94 | **Maternal Vaccination Messaging: An Analysis of Patient‐Facing Web‐Based Informatics Using Advanced Data Scraping Technologies**

Marta Szymanska^1^; Hanna R. Perone^2^; Kara Patek^3^; Bernard Gonik^4^

^1^Corewell Health West, Holland, MI; ^2^Detroit Medical Center, Detroit, MI; ^3^Wayne State University Detroit Medical Center, Detroit, MI; ^4^Division of Maternal Fetal Medicine, Department of Obstetrics and Gynecology, Wayne State University, Detroit, MI

**Objective**: Maternal vaccination (MV) protects pregnant individuals and their infants from vaccine‐preventable diseases. Suboptimal uptake persists due to barriers such as inadequate and mis‐information. Internet‐based informatics, such as digital health platforms, are readily accessible and can be transformative or conversely may negatively impact MV.   This study explores web‐based digital access points to MV information to assess related messaging.

**Study Design**: Using pre‐defined terms, a Google search identified 5000 potentially relevant English language websites related to MV. After relevance filtering, 2317 websites were included for analysis. Python programming applied the Flesch‐Kincaid readability formula, an automated 4‐point bias scoring system, and a pre‐designated lexicon of emotion‐generating terms for study.  Data were sub‐analyzed by domain type.  A random subset of sites underwent manual JAMA bias scoring to assess reliability of the automated bias score. Statistical analysis utilized ANOVA, Kruskal‐Wallis, and chi‐squared testing.

**Results**: The average website reading level was grade 11.7 (SD 2.6), with most exceeding standards for optimal readability. The mean bias score was 3.0 (SD 1.0), with frequent omissions of key transparency elements.  Results differed by domain type with  .com sites the most readable but the least transparent, while  .gov domains were more transparent but harder to read.  Emotion‐generating terms were detected across domains contributing to elevated emotional tone in content. All domain‐based differences were statistically significant (*p* < 0.001).

**Conclusion**: MV content online is difficult to read, lacks key transparency elements, and contains emotionally charged language. Commercial websites are more readable but omit critical sourcing, while  .gov and  .edu domains are more transparent but less understandable. These findings likely compromise informed patient decision‐making. Optimizing digital MV information for readability, transparency, and emotional neutrality is essential to support MV confidence and equitable uptake.

**9:15 AM – 9:30 AM**

## 95 | **Virtual Maternity Program Engagement and Neonatal Outcomes: A Medicaid Dose‐Response Analysis**

Lena Bertozzi^1^; Tanya Rudakevych^1^; Thomas Galeon^1^; Adam Kubsh^1^; Madeline Perry^2^; Isabelle Von Kohorn^1^; Ian J. Hooley^1^

^1^Pomelo Care, Pomelo Care, NY; ^2^University of Pennsylvania, University of Pennsylvania, PA

**Objective**: To evaluate the association between duration of prenatal engagement with a virtual maternity program and Neonatal Intensive Care Unit (NICU) and preterm birth outcomes in a six‐state Medicaid population.

**Study Design**: This retrospective claims analysis (1/1/2024‐3/31/2025) assessed NICU average length of stay (ALOS), NICU admission rates, NICU costs, and preterm birth as primary outcomes. Engagement duration (1+, 2+, or 3+ months) with the virtual maternity program was the primary exposure. Inverse probability weighting and double‐robust regressions adjusted for demographics, prior healthcare use and outcomes, and medical conditions.

**Results**: Among 6630 patients engaged for 1+ months, NICU ALOS decreased by 11.48% (*p* < 0.05), NICU admissions by 4.08% (*p* > 0.1), NICU cost by 7.36% (*p* > 0.1), and preterm birth increased by 2.67% (*p* > 0.1) (Table 1). For 4,550 patients engaged for 2+ months, NICU ALOS decreased by 15.56% (*p* < 0.05), NICU admissions by 6.40% (*p* > 0.1), NICU cost by 9.92% (*p* < 0.1), and preterm birth decreased by 9.59% (*p* < 0.1). For 3008 patients engaged for 3+ months, NICU ALOS decreased by 26.80% (*p* < 0.001), NICU admissions by 12.56% (*p* < 0.05), NICU cost by 17.40% (*p* < 0.01), and preterm birth decreased by 18.84% (*p* < 0.01). These reductions can be attributed to the program's direct medical care, including 24/7 remote management of conditions, mental health support, social needs support, and continuous care coordination.

**Conclusion**: Lengthy NICU stays create significant financial and emotional burdens, especially for underserved populations. This study demonstrates a significant inverse dose‐response relationship between virtual maternity program engagement duration and NICU ALOS, admissions, costs, and preterm birth rates in a multi‐state Medicaid cohort. Longer engagement is associated with greater reductions and statistical significance, suggesting these programs can improve neonatal outcomes and mitigate prolonged NICU admissions and associated costs.

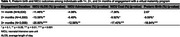

**9:30 AM – 9:45 AM**

## 96 | **Non‐Birthing Partner Outcomes in a Randomized Controlled Trial of the Baby2Home Digital Mental Health Intervention**

Emily S. Miller^1^; Jacqueline Gollan^2^; Lutfiyya N. Muhammad^3^; Kathleen O'Sullivan^2^; Dinah Williams^4^; Malika D. Shah^3^; Lynn M. Yee^3^; Siyuan Dong^3^; Young S. Lee^3^; Craig F. Garfield^2^

^1^Women & Infants Hospital of Rhode Island / Warren Alpert Medical School of Brown University, Providence, RI; ^2^Northwestern University Feinberg School of Medicine, Evanston, IL; ^3^Northwestern University Feinberg School of Medicine, Chicago, IL; ^4^Women & Infants Hospital of Rhode Island, Providence, RI

**Objective**: The well‐being of non‐birthing partners supports maternal mental health, family functioning, and infant development. Nevertheless, few digital health interventions have been designed to include non‐birthing parents. We evaluated the effect of Baby2Home (B2H), a digital health intervention, on mental health, relationship wellness, and self‐efficacy of non‐birthing parents over the first postpartum year.

**Study Design**: In a randomized controlled trial enriched with dyadic participation, 642 postpartum families were assigned to B2H or usual care. B2H, a digital health tool grounded in the collaborative care model, includes co‐parent communication tools, parenting education, and mental health support over the first 12 months postpartum. With birthing participant approval, partners could enroll and were assigned to the same intervention arm. Patient‐reported outcomes were collected at 1, 2, 4, 6, and 12 months postpartum. Outcomes included stress (PSS), depression (PHQ9), anxiety (GAD7), global health (PROMIS‐GH), relationship satisfaction (RDAS), and self‐efficacy (PROMIS‐SE). Linear mixed‐effects models estimated between‐group differences.

**Results**: Among the 642 birthing participants, 324 (50.5%) partners consented to participate (B2H *n* = 151, usual care *n* = 173). Non‐birthing participants randomized to B2H reported significantly lower stress (β –2.72, 95% CI –4.13, –1.32) compared to usual care (Figure). Depressive symptoms were lower in the B2H group, though the difference did not reach statistical significance (β –0.68, *p* = 0.07). No significant differences were observed in anxiety (β –0.15, *p* = 0.69), global health (β –1.26, *p* = 0.33), relationship satisfaction (β 1.08, *p* = 0.17), or self‐efficacy (β 0.34, *p* = 0.64).

**Conclusion**: The B2H intervention significantly reduced stress among non‐birthing parents, though no significant differences were observed for other patient‐reported outcomes. Evidence‐based digital tools such as B2H supporting non‐birth partner well‐being may be a pathway to improve the health of the family unit, though further refinement is needed to optimize effectiveness.

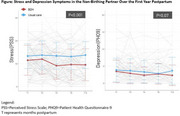

**9:45 AM – 10:00 AM**

## 97 | **Maternal Outcomes After Implementation of a State Medicaid Agency and Hospital System Partnership Care Model**

Catherine Eppes^1^; Suzanne Lundeen^2^; Ashley Sperber^2^; Sarah Detlefs^1^; Catherine Gannon^1^; Michelle Hansford^3^; Vaughan Gilmore^4^; Abigail Candelari^1^; Nidal Moukaddam^1^; Andres Ojeda^1^

^1^Baylor College of Medicine, Houston, TX; ^2^Harris Health System, Houston, TX; ^3^Santa Maria Hostel, Housotn, TX; ^4^Santa Maria Hostel, Houston, TX

**Objective**: Substance Use Disorder (SUD) is one of the leading causes of maternal morbidity and mortality throughout the United States.  We implemented a Maternal Opioid Misuse Model (MOM) pilot program via a partnership between a safety net hospital system and Texas Medicaid agency designed to optimize maternal outcomes related to SUD.

**Study Design**: The Center for Medicaid Service funded the MOM program from 2020‐2025.  The program goals included improving quality of care, reducing cost and increasing access to treatment services.  Model design key features (Figure 1) include: universal screening and referral, integrated medical, behavioral health and community services, targeted doula and navigation support, individualized pain management strategies, “Care by Parent Suites” for an Eat Sleep Console program and implementation of a Plan of Safe Care.   Demographics and outcome data were collected for women with OUD in MOM and compared to similar populations in other studies, MMRC data, and hospital level data.

**Results**: 135 pregnancies were enrolled in the program from 2022–2025 who reported high rates of physical, emotional or sexual abuse (74%) and health related social needs (78%).  The majority were on MOUD (85%), including methadone (36%) and Buprenorphine (65%). Outcome data are included in Table 1. Universal screening for SUD increased, and 2% of hospital patients screened positive for being at risk for complications related to SUD.  Maternal‐infant dyad separation decreased from 68% to 15%. Diagnosis of neonatal opioid withdrawal syndrome (NOWS) decreased from 58% to 14. The NICU length of stay decreased from a historic average of 16 to 19 days to 4 days.

**Conclusion**: Integrated care models between health care systems and state Medicaid agencies have the potential to improve outcomes for pregnant patients with opioid use disorder.